# Predictive value of anti-Mullerian hormone for pregnancy outcomes following assisted reproductive techniques (ART) in Southwest China

**DOI:** 10.1186/s12978-022-01524-5

**Published:** 2022-12-13

**Authors:** Ling Liu, Xing-Yu Sun, Huan Yang, Xin-Jian Feng, Yun-Zhu Lan

**Affiliations:** 1 Department of Reproduction, The Affiliated Hospital of Southwest Medical University, Luzhou, Sichuan China; 2 Department of Gynecology, Shenzhen Second People’s Hospital/The First Affiliated Hospital of Shenzhen University Health Science Center, Shenzhen, Guangdong China; 3 Luzhou People’s Hospital, Luzhou, Sichuan China

**Keywords:** AMH, Assisted reproductive techniques, Embryo quality, Pregnancy outcomes

## Abstract

**Background:**

Anti-Müllerian hormone (AMH) is secreted by granulosa cells in preantral follicles and small antral follicles. There is limited information about whether serum AMH levels are related to pregnancy outcomes during in vitro fertilization and embryo transfer (IVF-ET). The aim of this study was to provide a theoretical basis for improving pregnancy outcomes.

**Methods:**

A retrospective cohort study was conducted on infertile women who were treated at the Reproductive Centre of the Affiliated Hospital of Southwest Medical University between September 2018 and September 2019. The sample included 518 participants from Southwest China. The participants were divided into 2 groups according to their AMH level. Their data were retrieved from the medical records: days and dosage of gonadotropin (Gn) (one bottle equals 75 IU), the number of oocytes obtained, the number of oocytes in metaphase II (MII) and the number of high-quality embryos. The pregnancy outcomes were followed up and divided into two groups according to whether they were pregnant or not, with statistical analysis of the parameters related to the in vitro fertilization process performed separately.

**Results:**

Compared to a lower AMH level (AMH ≤ 1.1), a higher AMH level (AMH > 1.1) resulted in less total Gn (bottle) (P = 0.00 < 0.05) and a lower starting Gn (IU) (P = 0.00 < 0.05), while the number of oocytes obtained,MII,cleavages and high-quality embryos were higher (P = 0.00 < 0.05). The participants' pregnancy outcomes (ectopic pregnancy, miscarriage, singleton, twin, multiple births) were found to not be predictable by AMH through ROC curves (P = 0.980, 0.093, 0.447, 0.146, 0.526, and 0.868 > 0.05). For participants in the pregnancy group, although AMH was lower in the nonpregnant participants(P = 0.868 > 0.05), the difference was not statistically significant, and the correlation coefficients between the two groups suggested no differences in the IVF process, except for the starting Gn (IU) (P = 0.038 < 0.05).

**Conclusion:**

AMH has clinical application value in predicting ovarian reserve function, providing guidance and suggestions for the specific formulation of ovulation promotion programs with assisted reproductive technology, but it cannot effectively predict the outcome of clinical pregnancy.

## Plain language summary

Compared with traditional parameters, including FSH, LH, E2, INH-B and AFC, for predicting outcomes of pregnancy during ART, AMH has good repeatability, strong stability and little fluctuation during different periods of the menstrual cycle, which has stronger clinical application value. However, there is limited information about whether serum AMH levels are related to pregnancy outcomes after in vitro fertilization and embryo transfer (IVF-ET). This research examined the ability of AMH levels to predict pregnancy outcomes following assisted reproductive techniques. A retrospective cohort study on 518 infertile women treated at the Affiliated Hospital of Southwest Medical University between September 2018 and September 2019 was conducted. The higher AMH (AMH > 1.1) group had less total Gn (bottle) (35 (25–44) vs. 42 (36–50.8), P = 0.00 < 0.05) and a lower starting Gn (IU) ((225 (150–300) vs. 300 (225–300), P = 0.00 < 0.05), while the numbers of oocytes obtained,the numbers of MII,the numbers of cleavages and the numbers of high-quality embryos were greater (P = 0.00 < 0.05).The participants' pregnancy outcome (ectopic pregnancy, miscarriage, singleton, twin, multiple births) could not be predicted by AMH as shown through ROC curves (P > 0.05). For participants in the pregnancy group, the difference in the starting Gn (IU) was statistically significant (IU) (225 (150–300) vs. 225 (187.5–300), P = 0.038 < 0.05). Although AMH was lower in the nonpregnant participants (3.630 (1.940–6.110) vs. 3.750 (1.960–5.885), P = 0.868 > 0.05), the difference was not statistically significant, and the correlation coefficients between the two groups suggested no difference in the IVF process.

These findings suggest that AMH has clinical application value in predicting ovarian reserve function, providing guidance and suggestions for the specific IVF protocol, but it cannot effectively predict the outcome of clinical pregnancy.

## Abstract (de)

**Hintergrund:**

Anti-Müllerian-Hormon (AMH) wird von Granulosazellen in präantralen Follikeln und kleinen Antralfollikeln sezerniert. Es gibt nur wenige Informationen darüber, ob die AMH-Serumspiegel mit den Schwangerschaftsergebnissen bei In-vitro-Fertilisation und Embryotransfer (IVF-ET) zusammenhängen. Das Ziel dieser Studie war es, eine theoretische Grundlage für die Verbesserung der Schwangerschaftsergebnisse zu schaffen

**Methoden:**

Es wurde eine retrospektive Kohortenstudie an unfruchtbaren Frauen durchgeführt, die zwischen September 2018 und September 2019 im Reproduktionszentrum des Affiliated Hospital der Southwest Medical University behandelt wurden. Die Stichprobe umfasste 518 Teilnehmerinnen aus dem Südwesten Chinas. Die Teilnehmerinnen wurden nach ihrem AMH-Wert in 2 Gruppen eingeteilt. Ihre Daten wurden aus den Krankenakten entnommen: Tage und Dosierung von Gonadotropin (Gn) (eine Flasche entspricht 75 IE), die Anzahl der gewonnenen Eizellen, die Anzahl der Eizellen in der Metaphase II (MII) und die Anzahl der hochwertigen Embryonen. Die Schwangerschaftsergebnisse wurden nachverfolgt und in zwei Gruppen eingeteilt, je nachdem, ob sie schwanger waren oder nicht, wobei die statistische Analyse der Parameter im Zusammenhang mit der In-vitro-Fertilisation getrennt durchgeführt wurde.

**Ergebnisse:**

Im Vergleich zu einem niedrigeren AMH-Spiegel (AMH≤1.1) führte ein höherer AMH-Spiegel (AMH>1.1) zu einer geringeren Gesamt-Gn (Flasche) (P=0.00<0.05) und einer geringeren Start-Gn (IU) (P=0.00<0.05), während die Anzahl der gewonnenen Eizellen, MII, Spaltungen und Embryonen hoher Qualität höher waren (P=0,00<0,05). Die Schwangerschaftsergebnisse der Teilnehmerinnen (Eileiterschwangerschaft, Fehlgeburt, Einlings-, Zwillings- und Mehrlingsgeburten) ließen sich anhand der ROC-Kurven nicht durch AMH vorhersagen (P=0.980, 0.093, 0.447, 0.146, 0.526 und 0.868>0.05). Bei den Teilnehmerinnen der Schwangerschaftsgruppe war der AMH-Wert bei den nicht schwangeren Teilnehmerinnen zwar niedriger (P=0.868>0.05), aber der Unterschied war statistisch nicht signifikant, und die Korrelationskoeffizienten zwischen den beiden Gruppen deuteten auf keine Unterschiede im IVF-Prozess hin, mit Ausnahme des Start-Gn (IU) (P=0.038<0.05).

**Schlussfolgerung:**

AMH hat einen klinischen Anwendungswert bei der Vorhersage der ovariellen Reservefunktion und liefert Anhaltspunkte und Vorschläge für die spezifische Formulierung von Ovulationsförderungsprogrammen mit assistierter Reproduktionstechnologie, kann aber das Ergebnis der klinischen Schwangerschaft nicht effektiv vorhersagen.

## Background

With the emergence of later marriage and childbearing, more accurate and intuitive requirements are being asked for during fertility evaluation. At present, indicators for evaluating ovarian reserve function include age, follicle stimulating hormone (FSH), luteinizing hormone (LH), oestradiol (E2), antral follicle (AFC) and anti-Müllerian hormone (AMH). Among these, AMH is secreted by granulosa cells of preantral follicles and small antral follicles, inhibits the recruitment of primordial follicles, regulates the growth and development of follicles [1]**,** is not regulated by the hypothalamus-pituitary-ovarian axis (HPO), affects the continuous growth of antral follicles [2], and can be detected on any day of the menstrual cycle [3]. Generally, AMH is stably maintained throughout the menstrual cycle [4]. Therefore, AMH is considered to be the best indicator to evaluate ovarian reserve.

For infertile women, AMH, which is volatile, can indicate changes in ovarian function, which is detectable earlier and is more accurate than traditional methods [5].Previous studies have shown that AMH is a good biomarker for the prediction of pregnancy outcome**s **[6, 7]. A meta-analysis which indicated a weak albeit a significant association between AMH and implantation with AUC of 0.591, and 52.2% sensitivity and 61.1% specificity [8].However, there is another view that the basal AMH level cannot predict the pregnancy outcome of IVF [9],and in no situation does AMH reflect oocyte health or chances for conception [10]. These findings are contradictory and deserve further study.

Herein, this study explored the statistical significance of the correlation factors of different AMH groupings during the IVF process and explored the value of AMH in predicting pregnancy outcomes using ROC curves to retrospectively analyse the clinical data of the IVF process.

## Methods

### Patient population

This is a retrospective study of infertile women who were treated at the reproductive centre of the Affiliated Hospital of Southwest Medical University between September 2018 and September 2019. This study was approved by the Institutional Review Board and the Ethics Committee of the Affiliated Hospital of Southwest Medical University. We estimated the required sample size based on the “multistage random sampling survey”. Inclusion criteria: menstrual regularity (the menstrual period was 3–7 days, the menstrual cycle was 24 to 35 days, self test body temperature or B ultrasound could be used to detect ovulation), both ovaries were present and had not been treated with gonadotropin for at least 6 months, a body mass index (BMI) between 19 and 30 kg/m2, and ovarian stimulation used a GnRH agonist (GnRHa)-long protocol. participants with metabolic abnormalities or endocrine diseases (such as polycystic ovary syndrome, diabetes, abnormal thyroid function, Cushing syndrome, etc.), chromosomal abnormalities in either the participant or the male partner, a history of ovarian surgery, infectious diseases, autoimmune diseases, allergic diseases, tumours, hepatitis and other diseases, a history of thrombosis or family history, and endometriosis were excluded.

Selected infertile participants who were undergoing IVF/ICSI-ET had their data extracted from the medical records: AMH, the total amount of gonadotropin (Gn) (bottles: one bottle equals 75 IU), start Gn (IU), the total days of Gn, the numbers of oocytes obtained,the numbers of oocytes in metaphase II (MII),the numbers of cleavages,the numbers of 2PN,the numbers of high quality embryos and the clinical pregnancy status. The final study sample size was 518.

### AMH measurement

Collection of blood samples occurred between 7 and 10 am with the extraction of 5 ml of fasting elbow venous blood. It was centrifuged at 1200*g* for 10 min at room temperature and the AMH level was detected by ELISA (AMH quantitative test kit, Guangzhou Kangrun Biotechnology Co., Ltd. Product number KR-AMH-001, a three-step double antibody sandwich ELISA quantitative detection method. The minimum detectable AMH concentration was 0.06 ng/ml. The variation parameter CV of the detection concentration among the three batch number kits was ≤ 15% (n = 10)). A DNM-9602 microplate reader was used for detection. The experimental procedures were performed strictly in accordance with the kit and instrument instructions.

### Controlled ovarian stimulation protocol

All women underwent IVF/ICSI cycles using a GnRH agonist (GnRHa) long protocol. Serum progesterone levels were measured at Days 19–23 of menstruation and when progesterone > 5 ng/ml, short-acting GnRHa (Triprelin Acetate Injection, Changchun Jinsai Pharmaceutical Co., Ltd.) 0.1 mg subcutaneous injection was given once a day for inhibition of pituitary function. At 14–17 days, B-ultrasound was used to measure the endometrial thickness and follicle size, and serum LH, FSH, and E2 were measured (endometrial thickness ≤ 5 mm, follicular size < 5 mm: LH, FHS ≤ 5 mIU/ml, E2 ≤ 20 pg/ml).

The Gn drugs were chosen according to the participant’s economic level (Gonna-f, Urofolotropin, Merck. Jinsaiheng, Lizhu group Lizhu pharmaceutical factory) and after starting Gn, vaginal B ultrasound was used for detection of follicular regulation by the Gn dosage. When the follicular diameter was 18–22 mm, an HCG trigger (recombinant human choriogonadotrophin alfa solution for injection, Merck, commercial name Aize) was injected that night. After 36–38 h, eggs were collected under transvaginal B-ultrasound by qualified surgeons. According to the semen quality, in vitro fertilization or intracytoplasmic sperm microinjection was used for fertilization, and high-quality embryos were selected for transfer after 3 days.

### Determination of fertilization

According to the development and morphological evaluation of the oocyte, the oocyte is divided into three classes: mature oocytes (MII stage): the first polar body can be seen in the perivitelline space, and the foaming of the oocyte cytoplasm disappears; intermediate mature oocytes (MI phase): the perivitelline space is not seen in the polar body; immature oocytes (GV stage): germinal follicles can be seen in the cytoplasm [11]. The mature oocytes obtained in this study were at the MII stage. Normal fertilization refers to two pronuclear (PN) and two polar bodies (polar body 2, Pb2) under a microscope, while abnormal fertilization refers to ≤ 1 (monopronuclear, 1PN) or ≥ 3 pronuclear (multipronuclear, 3PN).

According to the Peter cleavage stage [12] embryo scoring system, embryo quality was evaluated according to the size, shape, debris and cytoplasmic granules of cleavage balls. Class I and II embryos that developed from Day 3 to 6–10 cleavage balls were defined as high-quality embryos, and the others were defined as low-grade embryos.

The following data were collected: medical records, age, total dosage (bottles), days of gonadotropin (Gn), starting dose of gonadotropin (bottles), the number of oocytes obtained, the number of oocytes in metaphase II (MII) and the number of high-quality embryos. The primary outcome measure was the clinical pregnancy. Clinical pregnancy was defined as the presence of a gestational sac with foetal heartbeat detection by ultrasonography.Ectopic pregnancy is included in the record when the first ultrasound suggests an extrauterine pregnancy. Miscarriages mainly included: biochemical pregnancy was defined as a blood HCG greater than 25 U/L or a positive HCG test in urine but no gestational sac on ultrasound, early miscarriage was defined as a follow-up ultrasound indicating no yolk sac or cessation of embryonic development from 28 days to 12 weeks post-transplantation, and late miscarriage was defined as 12 weeks to 28 weeks gestation, and this study was followed up to the final outcome of the patient post-transplantation.

### Statistical analysis

SPSS 17.0 was used for statistical analysis, and all data were first tested for normality; as the data of AMH, the total gonadotropin (Gn) (bottles: One bottle equals 75 IU), start Gn (IU), the total days of Gn, the numbers of oocytes obtained, the numbers of oocytes in metaphase II (MII), the numbers of cleavages,the numbers of 2PN and the numbers of high quality embryos were skewed, we used median and interquartile ranges to describe the data, applying a nonparametric test (Mann–Whitney test). A value of p < 0.05 was considered statistically significant. The optimal regression model was selected to analyse the smooth fitting curve. ROC curve analysis was performed to determine whether AMH could predict pregnancy outcomes. Graph Prism was used to plot pie charts, histograms and scatter plots. p < 0.05 suggested statistical significance.

## Results

### Comparison of clinical outcomes of IVF or ICSI in the two groups

**Fig. 1:**
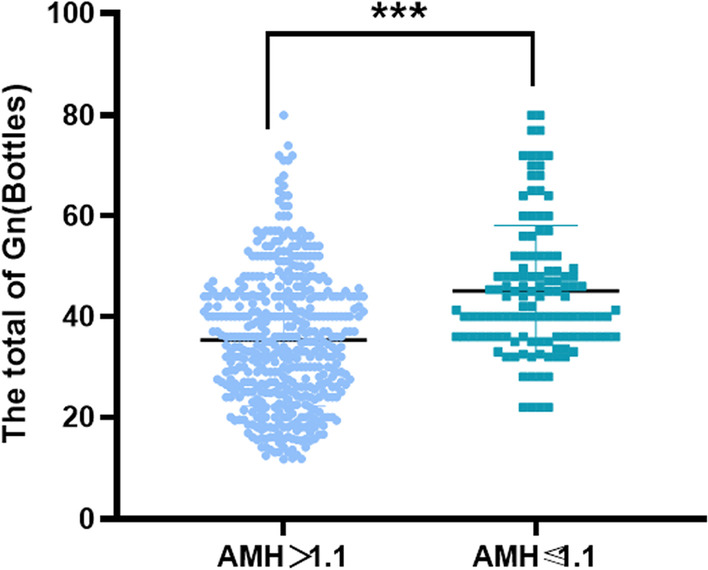
Scatter plot of the total of Gn (bottles)

**Fig. 2:**
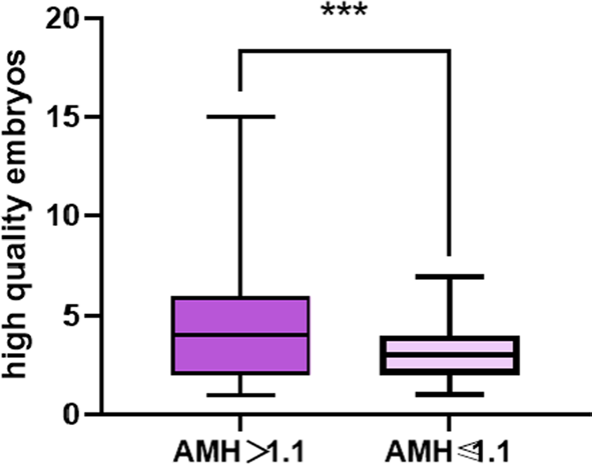
Box diagram of high-quality embryos

The 518 participants included in this study were divided into those with high AMH (AMH > 1.1 ng/ml) (n = 457) and low AMH (AMH ≤ 1.1 ng/ml) (n = 61). The baseline characteristics are presented in Table 1. The variables were skewed, so we used median and interquartile ranges to summarize the data. It can be seen that the high AMH group used less Gn (bottles) (35 (25–44) vs. 42 (36–50.8), P = 0.00 < 0.05) and less starting Gn (IU) ((225 (150–300) vs. 300 (225–300), P = 0.00 < 0.05), and the numbers of oocytes obtained,the numbers of MII,the numbers of cleavages and the numbers of high quality embryos were higher (P = 0.00 < 0.05). The scatter plot of the total Gn (bottles) is shown in Fig. 1, and the box diagram of high-quality embryos is shown in Fig. 2.

**Table 1:** Comparison of clinical outcomes of IVF or ICSI between the two groups

	A1 group (n = 457) AMH > 1.1 ng/ml	A2 group (n = 61) AMH ≤ 1.1 ng/ml	Z	P
The total Gn (bottles)	35 (25–44)	42 (36–50.8)	− 4.959	0.000
Start Gn (IU)	225 (150–300)	300 (225–300)	− 6.349	0.000
The total days of Gn	11 (10–12)	11 (9–12.5)	− 0.542	0.588
Number of oocytes obtained	10 (8–13)	6 (4–7)	− 8.342	0.000
The number of MII	9 (7–12)	5 (3–6.5)	− 7.705	0.000
The number of cleavages	8 (5–11)	5 (3–6)	− 7.119	0.000
The number of 2PN	6 (4–9)	4 (2–5)	− 5.978	0.000
The number of high quality embryos	4 (2–6)	3 (2–4)	− 4.501	0.000

### AMH predicts pregnancy outcomes

**Fig. 3:**
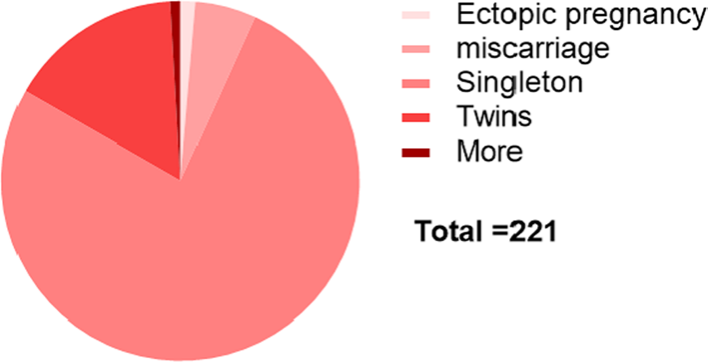
Pie chart of pregnancy outcomes

To determine whether AMH has a predictive value for pregnancy outcomes, by following up the pregnancy outcomes of IVF,participants, including ectopic pregnancy, miscarriage, singleton, twins and multiple births, using ROC curves, we found that AMH may not have a good predictive value for pregnancy (Fig. 3) outcomes of IVF (P = 0.980, 0.093, 0.447, 0.146, 0.526, > 0.05) (see Table 2).

**Table 2:** The predictive effect of AMH for ectopic pregnancy, abortion, singleton, twins and more than three foetuses in the pregnancy group

Results	Area under ROC curve	SE	P	Lower limit of area under ROC curve	Upper limit of area under ROC curve
Ectopic pregnancy	0.496	0.117	0.980	0.266	0.726
miscarriage	0.642	0.088	0.093	0.469	0.814
Singleton	0.477	0.031	0.447	0.416	0.538
Twins	0.574	0.050	0.146	0.475	0.672
multiple births	0.370	0.229	0.526	0.000	0.820

### Comparison between the pregnancy group and the nonpregnancy group

**Fig. 4:**
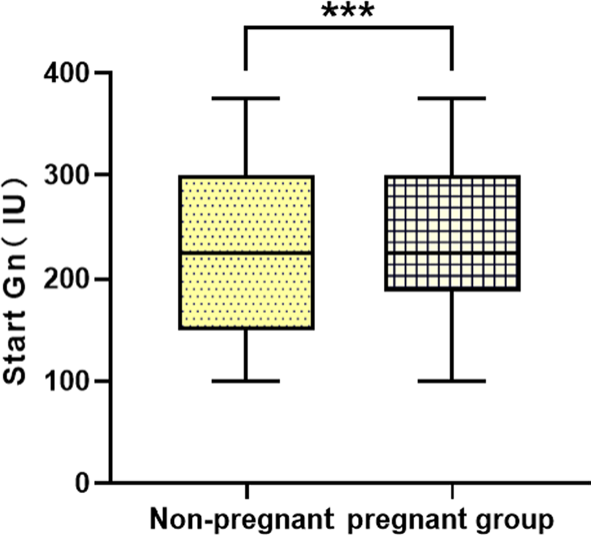
Box diagram of start Gn (IU)

We included 518 participants who were divided into a pregnancy group (n = 221) and a nonpregnancy group (n = 297),Pregnancy rate is 42.66%,as shown in Table 3, the starting gonadotropins dose was significantly higher in the pregnancy group than in the nonpregnancy group (225 (150–300) vs. 225 (187.5–300), P = 0.038 < 0.05)(see Fig. 4). Other variables, including the total gonadotropin dose, total days of use of gonadotropin,the numbers of oocytes retrieved, the numbers of MII,the numbers of cleavages,the numbers of 2PN and numbers of good quality embryos, showed no differences between the two groups.

**Table 3:** Comparison between the pregnancy groupand the nonpregnancy group

	Nonpregnant group (n = 297)	Pregnant group (n = 221)	Z	P
AMH	3.630 (1.940–6.110)	3.750 (1.960–5.885)	− 0.166	0.868
The total Gn (bottle)	36 (25–46)	36 (27–44)	− 0.379	0.705
Start Gn (IU)	225 (150–300)	225 (187.5–300)	− 2.073	0.038
The total days of Gn	11 (10–12)	11 (10–12)	− 0.497	0.619
Number of oocytes obtained	10 (7–13)	10 (7–13)	− 0.359	0.720
The number of MII	9 (6–12)	9 (6–12)	− 0.534	0.593
The number of cleavages	7 (5–11)	8 (5–11)	− 0.300	0.764
The number of 2PN	6 (4–8)	6 (4–8.5)	− 0.389	0.697
The number of high quality embryos	4 (2–6)	4 (2–6)	− 1.015	0.310

## Discussion

Female reproductive function gradually changes throughout puberty, the growth period, the perimenopausal period and menopause, so the detection of ovarian function reserve is very important for clinicians to guide participants to choose fertility treatments and predict their clinical prognosis. Traditional ovarian function assessment methods include the determination of basic hormone levels (FSH, LH, E2), inhibin B (InhB), and antral follicles (AFC) [13]. Due to the complex interactions in the internal environment, FSH, InhB and AFC may not be able to predict the ovarian reserve function in women who do not have changes in ovulation. However, even for these women, AMH can still indicate changes in ovarian function, which is detectable earlier and is more accurate than traditional methods [14].

AMH is secreted by granulosa cells of preantral follicles and small antral follicles, inhibits the recruitment of primordial follicles, regulates the growth and development of follicles [15] and is not regulated by the hypothalamus-pituitary-ovarian axis. It stimulates the continuous growth of antral follicles, and it can be detected on any day during the menstrual cycle. AMH is produced by follicles entering the cycle and also by other follicles not entering the cycle, which can predict ovarian responsiveness and is not affected by FSH [16, 17]**.**

In a study of age in China, it was found that fertility tends to be static from the establishment of menarche to the age of 30 and it begins to decline after the age of 30. After the age of 50, the weight and volume of the ovaries decrease significantly, and ovarian function shows an exponential decay [18]. AMH could be reduced to 1.43 μg/L at age 37 and start to decline at 30 years old, especially after 35 years old. The level of AMH decreases linearly, which is positively correlated with AFC and the ovum capture rate [19].

During the process of assisted reproductive technology, the level of ovarian reserve function can affect the outcomes of ovulation and pregnancy. If a deficiency of ovarian reserve function can be detected before the implementation of assisted reproductive technology, we could deduce the results of drugs from the level of AMH and explore how to improve the clinical outcome. The sensitivity and specificity of AMH in evaluating ovarian hyporesponsiveness were reported to be 80% and 85%, respectively [20].In Iranian infertile women,researchers found the cutoff value of AMH with the prediction of poor response was 1.65 ng/ml with AUC of 0.8 (0.69–0.91),sensitivity was 89% and specificity was 71% [21].

Through this study, we can see that during the process of assisted reproductive technology, compared with low AMH (AMH < 1.1), the women with higher AMH (AMH > 1.1) used less Gn (bottle) (35 (25–44) vs. 42 (36–50.8)), had a lower starting Gn (IU) (225(150–300) vs. 300(225–300)), and during the oocyte acquisition and embryo culture, the numbers of oocytes obtained (10 (8–13) vs. 6 (4–7)), the numbers of MII (9 (7–12) vs. 5 (3–6.5)), the numbers of cleavages (8 (5–11) vs. 5 (3–6)), the numbers of 2PN(6 (4–9) vs. 4 (2–5), the numbers of high-quality embryos(4 (2–6) vs. 3 (2–4)) were all better (P < 0.001). This is similar to most other studies that found that serum AMH can better predict ovarian responsiveness and the numbers of oocytes retrieved during controlled ovarian hyperstimulation [22].

Compared with basic FSH, oestrogen and inhibin B and other indicators to predict ovarian reserve function in women, the stability of AMH, not affected by the detection time and with other advantages, makes it very useful in the field of assisted reproduction to evaluate ovarian reserve function and predict ovarian responsiveness and it has received a great deal of attention [23]**.** Is there any correlation between AMH and the clinical outcomes of assisted reproductive technologies? Interestingly, in our study, through ROC curves, we found that AMH may not have a good predictive value for pregnancy outcomes of IVF (P = 0.980, 0.093, 0.447, 0.146, 0.526, > 0.05). Although the AUC for miscarriage was equal to 0.642, indicating that the accuracy of AMH for predicting miscarriage as the IVF outcome could reach 64.2%, it was not statistically significant (P = 0.093, < 0.05).

Some researchers found that AMH could not only promote the development of oocytes but also increase the development potential of fertilized eggs [24]. However, there are researchers who found the opposite, that there is no correlation between basic AMH levels and pregnancy outcomes [25]and the age may increase the risk of infertility [26]. In this study, we included 518 participants who were divided into a pregnancy group (n = 221) and a nonpregnancy group (n = 297). We found a significant difference in the starting gonadotropin dose (start Gn) (225 (150–300) vs. 225 (187.5–300), P = 0.038 < 0.05). Other variables, including total gonadotropin dose, total days of use of gonadotropin, numbers of oocytes retrieved, numbers of MII, numbers of cleavages, numbers of 2PN and numbers of good-quality embryos, showed no differences between the two groups. We deduced that pregnancy outcome might be related to other variables that may improve the pregnancy outcome and increase the pregnancy rate in IVF participants, such as BMI, endometrium, and hysteroscopy status(In this study, these variables were not statistically different), which can be collected for analysis in future studies while increasing the sample size to reduce selection bias in data analysis.This study cannot be an accurate univariate analysis because it is a retrospective study; therefore, it can be built upon for future prospective designs to reduce bias in outcomes. However, it is worth acknowledging that this study can be predictive of starting Gn(bottles),Gn (bottles),the numbers of oocytes obtained,the numbers of MII,the numbers of cleavages and the numbers of high quality embryos by AMH for doctors when developing protocols.

## Conclusions

AMH can be used as a reference index to predict the ovarian reserve function of participants during the process of ovulation induction. More clinical sample accumulation and data analysis are needed to determine whether it is related to the clinical outcomes of assisted reproduction. During the process of IVF-ET research on participants, a multi-index joint test is a more accurate and objective method for predicting ovarian reserve function, which provides an individualized basis for clinicians to make decisions during the process of promoting ovulation. Early detection of ineffective treatment cycles reduces the economic burden on Infertile women, improves pregnancy outcomes, and improves pregnancy rates; thus, exploring the relationship between the two is our future research direction.

## Data Availability

There are no linked research data sets for this paper. Data will be made available on request.

## Abbreviations

ART: Assisted reproductive technology
AMH: Anti-Müllerian hormone
IVF-ET: In vitro fertilization and embryo transfer
MII: MetaphaseII
FSH: Follicle stimulating hormone
LH: Luteinizing hormone
E2: Oestradiol
AFC: Antral follicle
HPO: Hypothalamus-pituitary-ovarian axis
BMI: Body mass index
2PN: Two pronuclear
1PN: Monopronuclear
3PN: Multipronuclear
2Pb: Two polar bodies
CPR: Clinical pregnancy rate
Gn: Gonadotropin
ROC: Receiver operating characteristic curve
InhB: Inhibin B
